# “CAREGIVING IS TEAMWORK...” INFORMATION SHARING IN HOME CARE FOR OLDER ADULTS WITH DISABILITIES IN THE COMMUNITY

**DOI:** 10.1093/geroni/igad104.1198

**Published:** 2023-12-21

**Authors:** Chanee Fabius, Aleksandra Wec, Martha Abshire Saylor, Jamie Smith, Joseph Gallo, Jennifer Wolff

**Affiliations:** Johns Hopkins University, Baltimore, Maryland, United States; Johns Hopkins Bloomberg School of Public Health, Baltimore, Maryland, United States; Johns Hopkins University School of Nursing, Baltimore, Maryland, United States; Johns Hopkins University, Baltimore, Maryland, United States; Johns Hopkins University, Baltimore, Maryland, United States; Johns Hopkins University, Baltimore, Maryland, United States

## Abstract

Direct care workers provide assistance with daily activities to persons with disabilities. Direct care workers are well positioned to recognize and share information about care gaps and changes in function and behaviors but are often viewed as unskilled and excluded from interdisciplinary care team interactions. We conducted 11 semi-structured interviews with administrators (n=5), nurses (n=3), and direct care workers (n=3) from home care agencies (“residential service agencies” in Maryland) to understand information sharing between direct care workers, family caregivers, and clinicians in home care delivery for older adults with disabilities, including those living with dementia. Thematic analysis was completed by the research team. Leveraging Systems Engineering Initiative for Patient Safety (SEIPS) 2.0 model, a human factors framework, respondents highlighted aspects of information sharing across (1) work systems, (2) processes, and (3) dementia-related adaptations. Work system characteristics relevant to information sharing included the utilization of electronic management systems and patient portals to communicate within agencies and with clinicians and using multiple methods to gather information about client goals and preferences (e.g., assessments, client profiles). Process characteristics include collaborations between families and residential service agency staff to deliver optimal care. Direct care workers did not report differences in coordinating care for persons living with dementia, however they reported dementia-related adaptations like an increased need to establishing and sustaining strong relationships with family members and communicating with clinicians during medical visits. Findings highlight the care demands experienced by direct care workers and support calls to better coordinate information sharing between interdisciplinary care teams.

